# Improved Corrosive Resistance of Micro-Arc-Oxidation Coating on 6063 Aluminum Alloy by Co-Doping with Graphite and Sodium Tungstate

**DOI:** 10.3390/ma18040767

**Published:** 2025-02-10

**Authors:** Na Jia, Erhui Yang, Jianyang Zhu, Feiyan Liang, Weizhou Li, Xiuhai Zhang, Ruixia Yang

**Affiliations:** 1 School of Resources, Environment and Materials, Guangxi University, Nanning 530004, China; 18071394431@163.com (N.J.); 89yangeh@sina.com (E.Y.); zhujianyang5717@163.com (J.Z.); 19977470215@163.com (F.L.); wz-li@hotmail.com (W.L.); xiuhaizhang@gxu.edu.cn (X.Z.); 2 State Key Laboratory of Featured Metal Materials and Life-Cycle Safety for Composite Structures, Guangxi University, Nanning 530004, China; 3 School of Mechanical and Marine Engineering, Beibu Gulf University, Qinzhou 535011, China

**Keywords:** MAO, graphene oxide dispersion, Na_2_WO_4_, corrosion resistance

## Abstract

The present study investigates the effect of different concentrations of Na_2_WO_4_ and graphene oxide dispersed composite additives on the structure and corrosion resistance of 6063 aluminum alloy micro-arc oxidation (MAO) coatings in a silicate electrolyte. The characterisation of the microstructure, cross-sectional morphology, elemental distribution, and phase composition of the films was conducted utilising scanning electron microscopy (SEM), energy-dispersive spectroscopy (EDS), and X-ray diffraction (XRD). The corrosion resistance of the films was tested by prolonged immersion for 24 h, 72 h, 168 h, and 240 h, with measurement of kinetic potential polarisation curves and impedance modulus in a 3.5 wt.% NaCl solution. The densification of the films was enhanced with increasing mass concentration of Na_2_WO_4_ and dispersed graphene oxide in the electrolyte, and the thickness initially increased and then decreased. The film containing 6 g of Na_2_WO_4_ and 10 mL of graphene oxide dispersion (G10-6) exhibited optimal densification and thickness, with an I_corr_ value of 3.01 × 10^−6^ A·cm^−2^ and a low-frequency impedance film value of 10^8^ Ω·cm^2^, thereby demonstrating the most advanced corrosion resistance among the films. The densification and corrosion resistance of the films were enhanced by the incorporation of Na_2_WO_4_ and graphene oxide dispersion into the alkaline electrolyte.

## 1. Introduction

Aluminium and its alloys have excellent physical and chemical properties, including conductivity and thermal conductivity, atmospheric corrosion resistance [1], and processability. It is widely used in various fields such as automotive, aerospace, machinery manufacturing, construction, and electronics [2]. However, due to its low surface hardness, aluminium features low wear and corrosion resistance, which not only affects its appearance but also shorten its service life. Therefore, it is necessary to treat it against corrosion [3]. To overcome these weaknesses, many surface modification techniques for aluminium alloys have emerged. Among them, the commonly used surface strengthening methods for aluminium alloys include anodic oxidation [4], laser treatment [5], metal coating [6], vapour phase deposition [7], and micro-arc oxidation [8,9].

Micro-arc oxidation technology has received wide attention in recent years and has become a hot spot of research both domestically and internationally. Micro-arc oxidation, also known as anodic discharge deposition (ASD) [10], is a new process in which nonferrous metals and their alloys, such as aluminium, titanium, magnesium, etc., are placed in an electrolyte and a certain voltage or current is applied to form a micro-arc discharge on the surface of the workpiece. The high temperature and high pressure effect of micro-arc discharge is used to generate an oxide ceramic layer in situ on the metal surface [11]. Micro-arc oxidation technology has gradually become a common surface treatment method for aluminium alloys due to its advantages of high production efficiency, simple process, environmentally friendliness and non-polluting [12], strong bonding of the ceramic film with the metal substrate, and good electrical insulation and corrosion resistance [13]. However, the film layer generated by micro-arc oxidation technology inevitably contains pores and microcracks, which affect the corrosive resistance of the film layer. Meanwhile, it has been shown that the composition, concentration, and electrical parameters of the electrolyte in the micro-arc oxidation process will have a certain impact on the structure and performance of the film layer [14]. Among them, the electrolyte components are directly involved in the film-forming reaction process, which has a more significant impact on the performance of ceramic membranes and determines the composition and performance of ceramic membranes to a large extent. The results show that the addition of different kinds of additives to the electrolyte can effectively improve the performance of the micro-arc oxidation film. Among them, the addition of [15,16] particles with special functions such as ZrO_2_, CeO_2_, TiO_2_, as well as MoS_2_ nanoparticles [17], CeO_2_ [18], graphene [19], sericite microporous plates [20], and TiC [21] can improve the corrosive resistance of the micro-arc oxidation film. Chun chieh Tseng et al. [22] found that the silicate/hexametaphosphate system, by adding sodium tungstate, can effectively increase the Al_2_O_3_ content and increase the thickness of the oxide film, which is due to the increase in the micro-arc discharge voltage induced by sodium tungstate. Yajuan Liu et al. [23] found that, when 5 g/L Na_2_WO_4_ was added to the electrolyte, the results showed that the oxide coating contained WO_3_, the surface of the coating was smooth and dense, and the corrosion resistance was 1.6 times higher than that of the silicate coating. Li et al. [24] investigated the effect of sodium tungstate on the performance of the arc film layer of the AZ80 magnesium alloy in a silicate electrolyte system and found that sodium tungstate significantly increased the thickness and density of the film layer and improved the corrosion resistance of the film layer. Zuo et al. [25] used a micro-arc oxidation technique on a Ti6Al4V alloy for micro-arc oxidation treatment. The results showed that, with the increase in graphene oxide dosage, the electrolyte conductivity increased, porosity decreased, and hardness increased. The corrosion resistance of the coating was significantly improved when the addition amount was 10 mL. Askarnia et al. [26] also investigated the corrosion resistance of graphene oxide (GO) in the micro-arc oxidation coating of magnesium alloy. Their results showed that GO extends the zigzag conduction paths of corrosive media and provides exceptional barrier properties against these media, thus significantly improving the corrosion resistance of the metal substrate.

However, fewer studies have been reported on the preparation of micro-arc oxidation films by adding sodium tungstate and graphene oxide composite additives to the electrolyte. In order to obtain ceramic films with excellent film-forming properties and corrosion resistance, sodium silicate was used as the base electrolyte in this paper. Therefore, four different concentrations of sodium tungstate and graphene oxide dispersions were added as additives to form a novel composite electrolyte.

## 2. Experimental

### 2.1. Experimental Material

The 6063 aluminium alloy was selected as the matrix material, with a nominal composition (expressed as mass fraction) of Si 0.2%~0.6%, Mg 0.45%~0.9%, Fe ≤ 0.35%, Cu ≤ 0.1%, Mn ≤ 0.1%, Cr ≤ 0.1%, Zn ≤ 0.1%, Ti ≤ 0.1%, and Al balance. The dimensions of the specimen are 15 mm × 15 mm × 3 mm. The sample should be meticulously polished using waterproof sandpaper, gradually advancing to 2000-grit sandpaper. Following this, the sample should be rinsed with deionised water, then cleaned in an ultrasonic cleaning machine. It should then be dried with hot air and finally bagged for later use.

### 2.2. Preparation of MAO Paint

The micro-arc oxidation coating was produced using a pulsed power supply manufactured by China Yisheng Electronic Technology Co., Ltd. (Guangzhou, China). The equipment operated in a double-pulse constant voltage mode, with the oxidation process for the 6063 aluminium alloy sample taking place over a period of 25 min at a voltage of 400 V, a duty cycle of 15%, and a frequency of 400 Hz. The temperature of the circulating cooling system was maintained below 30 °C. The electrolytes employed for the MAO process comprised Na_2_SiO_3_ (10 g/L), NaF (2 g/L), and NaOH (2 g/L). Additionally, graphene oxide (10 mL) was incorporated into the mixture, along with varying amounts of Na_2_WO_4_—specifically, 4 g, 6 g, 8 g, and 10 g—to prepare five distinct samples, designated as G0, G10-4, G10-6, G10-8, and G10-10 for convenience. The composition of the various electrolytes is shown in Table 1.

### 2.3. Coating Characterisation

X-ray diffraction (MiniFlex600-C, XRD, Japan, nippon ryugaku corporation, Okazaki, Japan) was employed to investigate phase composition across a 2θ range from 10° to 85°. Cu Kα radiation (λ = 1.5418 Å) was utilised at a scanning rate of 4°/min, with a size set at 0.02°. The surface and cross-section of each coating were characterised using a scanning electron microscope (SEM, SU8020 Japan, Hitachi High-Technologies Co., Tokyo, Japan), and the compositions of the coating were analysed using the Japanese X-Max80 energy-dispersive spectroscopy (EDS, Hitachi High-Technologies Co.) system.

### 2.4. Film Corrosion Resistance Testing

The corrosion resistance performance was the focus of this study, which was conducted using an electrochemical impedance spectroscopy workstation (CS350, CorrTest, Wuhan, China) and potentiodynamic polarisation scanning curves. In a 3.5 wt.% NaCl aqueous solution, the working electrode was a micro-arc oxidation sample (exposed area of 1 cm^2^), the auxiliary electrode was a platinum electrode, and the reference electrode was a saturated calomel electrode (SCE). The amplitude was set to 10 mv and the frequency range was 10^5^–10^−2^ Hz. To study the long-term corrosion performance of the sample, EIS testing was conducted on the sample after soaking for 240 h, and the EIS data were analysed using Zview (3.1) software. The Tafel curve was fitted, and this provided the corrosion potentials E_corr_ (V) and the current densities I_corr_ (A·cm^−2^). The Stern Geary formula was then utilised to calculate the corrosion rate CR (mm·a^−1^).

(1) $$CR=\frac{MI_{\mathrm{corr}}}{n\rho F}$$

where M is the relative atomic mass of the metal in grams, n represents its valence electron, ρ represents the density of the metal (g/cm^3^), and F is Faraday’s constant.

## 3. Results

### 3.1. Coating Microstructure

**Figure 1:**
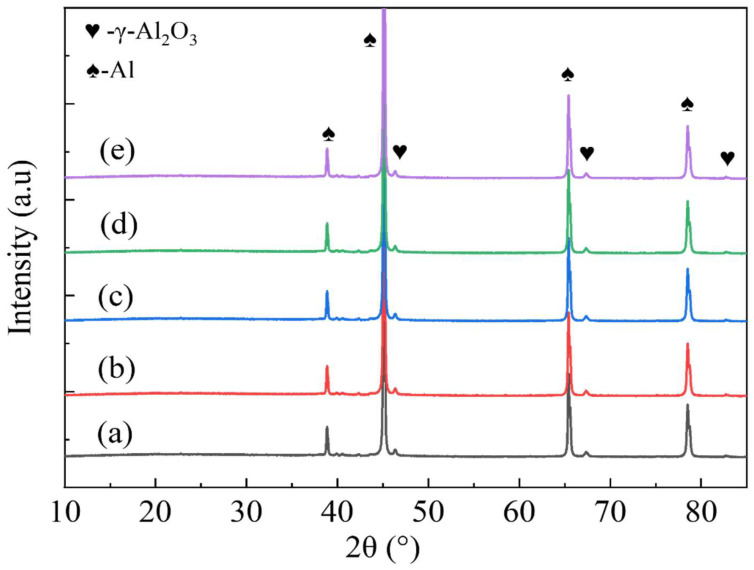
X-ray diffraction patterns of different micro-arc oxidation coatings: (a) G0, (b) G10-4, (c) G10-6, (d) G10-8, and (e) G10-10.

Figure 1 presents the XRD patterns of various MAO-coated samples. XRD analysis demonstrates the presence of a significant diffraction peak corresponding to Al in the 6063 aluminium alloy matrix on the surface of the ceramic coatings. This phenomenon can be attributed to the thinness of these ceramic coatings and the large number of micropores and microcracks on their surfaces; these features allow high-energy X-rays to penetrate in order to reach the substrate [27]. Furthermore, the presence of the γ-Al_2_O_3_ phase was identified within the micro-arc oxidised layer, as evidenced by the distinct diffraction peaks, suggesting a high degree of crystallinity in the film. The γ-Al_2_O_3_ content in the film layer remained relatively constant, irrespective of the composite additive concentration. However, there are no peaks corresponding to the elements C and W observed in the XRD patterns, which may be attributable to the low dispersion level of graphene oxide and sodium tungstate (≤10 g/L) resulting in a minimal amount of WO_3_ sufficiently reacting with abundant Al [28]. Furthermore, the coating’s thickness may be a contributing factor, with Na_2_WO_4_ present in the form of WO_3_/Na_2_WO_4_ or W elements, which are known to be involved in the formation of the coating, as previously reported [22].

### 3.2. Coating Morphology

**Figure 2:**
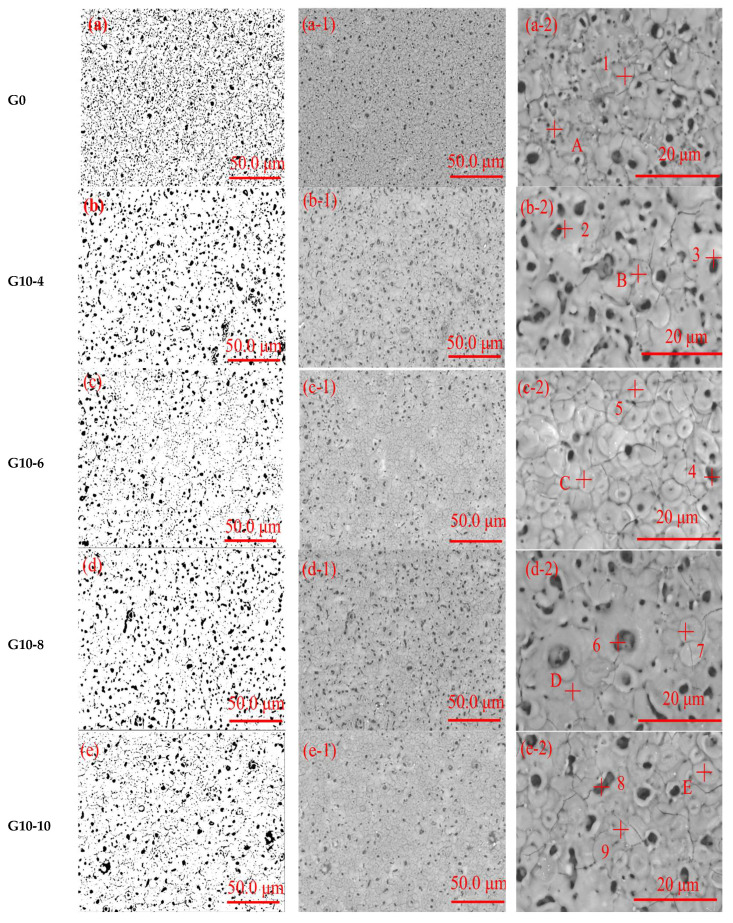
Micromorphology analysis of MAO coatings at different concentrations: (**a**) G0, (**b**) G10-4, (**c**) G10-6, (**d**) G10-8, and (**e**) G10-10.

As illustrated in Figure 2, the surface morphology and pore distribution of MAO coatings treated with graphene oxide dispersions and varying concentrations of Na_2_WO_4_ solution are evident. The results of the EDS analysis are presented in Table 2. The surface of the ceramic layer (Figure 2(a-1,a-2)) has a porous structure that is similar to that of “craters” and “volcanic piles”. This phenomenon can be attributed to the melting of the 6063 aluminium alloy matrix, a consequence of the elevated temperature encountered within the microdischarge channel. The molten aluminium then reacts with the electrolyte to form metal oxides, which are discharged outwards from the discharge channel under high pressure [29]. Assuming that the electrolyte temperature is maintained at approximately 30 °C, due to the rapid cooling effect of the electrolyte, these ejected molten metal oxides rapidly accumulate and solidify around the discharge microvia, resulting in the formation of a “crater”-like microscopic morphology on the surface of the ceramic layer (Figure 2(a-2) A). The porosity of the micro-arc oxidised coating was analysed using Image J software (Java 1.8.0_112 (64-bit)). The porosity was found to be approximately 15% ± 0.5 (Figure 2a). The micropores were observed to be surrounded by numerous microcracks, which extended towards the interior. This facilitated the penetration of corrosive media and reduced the protective properties of the micro-arc oxidation coatings, resulting in substrate corrosion. The EDS results at point 1 showed that the main elements on the surface of the pure MAO coatings were O and Al. With increasing concentrations of graphene oxide dispersion and Na_2_WO_4_ (Figure 2(b-1,c-1,d-1,e-1)). The surface of the film was found to be smooth, with a decrease in the number and size of pores, followed by an increase, and a decrease in porosity, followed by an increase. Some small pores on the surface of the ceramic layer in G10-4 (Figure 2(b-1,b-2)) were closed and the porosity decreased to 10.84% (Figure 2b). EDS analysis at points 2 and 3 detected 9.68 wt.% C and 3.12 wt.% w elements. It was observed that superior outcomes were achieved when G10-6 (Figure 2(c-1,c-2)) was utilised as the additive. Some of the large pores were closed, the coating surface was uniform and flat, and the porosity was reduced to 9.37% ± 0.5 (Figure 2c). The EDS of point 4 and 5 detected 4.74 wt.% C and 4.58 wt.% w elements, which is due to the fact that graphene oxide, which is uniformly dispersed in the electrolyte, absorbs free electrons and gradually migrates to the vicinity of the anode to be adsorbed on the surface of the sample under electrophoresis and forms a secondary discharge point of the micro-arc oxidation at its adsorption point, which significantly increases the number of discharge sparks. In addition, a small amount of molten oxide ejected from the inside of the discharge channel also covers the graphene oxide dispersion adsorbed on the sample surface. Under the rapid cooling effect of the electrolyte, it will condense on the bumps around the micropores [30] but in very small amounts. During the discharge breakdown, WO2− 4 enters the coating dense layer of the coating through the discharge channel. This is shown at points B, C, D, and E in Figure 2(b-2,c-2,d-2,e-2). This is consistent with the findings of Wang [31] et al. on the effect of nano-SiC on the microstructure of the surface of AZ91D magnesium alloy micro-arc oxide film. The addition of G10-8 (Figure 2(d-1,d-2)) and G10-10 (Figure 2(e-1,e-2)) additives increased the pore size of the coatings, and the porosity increased to 10.88% (Figure 2d) and 10.10% (Figure 2e), respectively. The EDS at points 6, 7, 8, and 9 showed a decrease in the elements C and W. The EDS at points 6, 7, 8, and 9 showed an increase in the pore size. Large holes and elongated cracks appeared near the holes in the film. This is due to the fact that excess sodium tungstate and graphene oxide prevented the holes in the ceramic film layer from closing efficiently [30]. It can be seen that 10 mL graphene oxide dispersion and 6 g of Na_2_WO_4_ solution can better fill the pores of the dense and loose layers in the MAO layer and play a good co-ordinating role.

**Figure 3:**
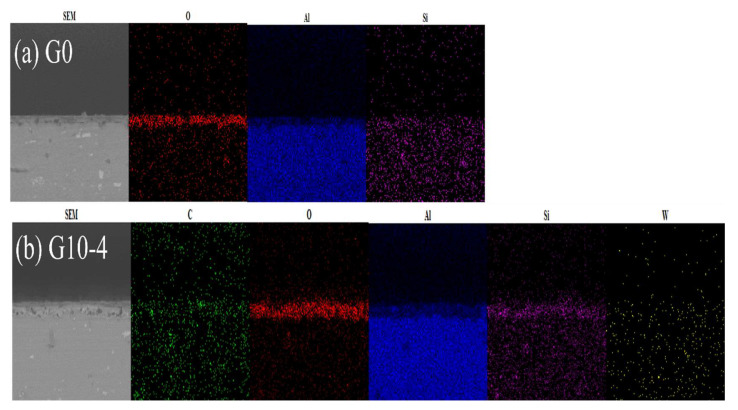

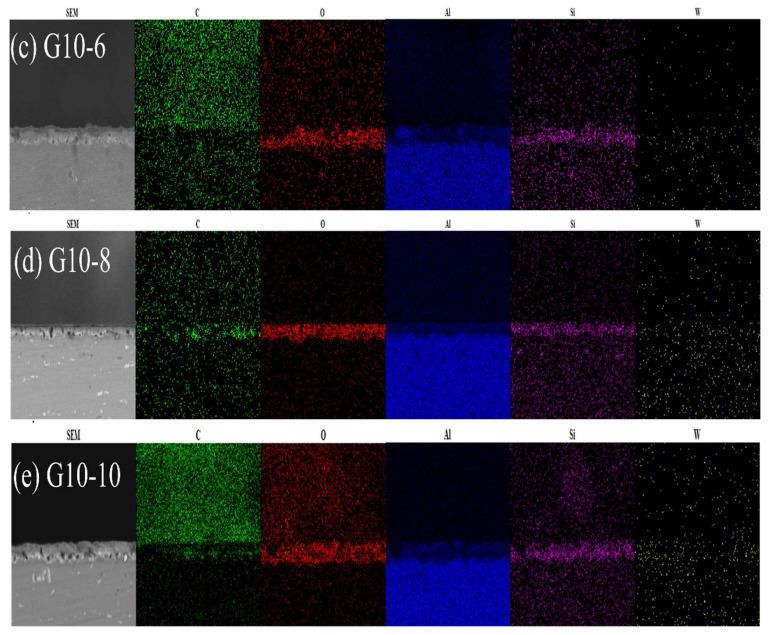
Surface scan analysis of micro-arc oxidation coatings with different concentrations.

**Figure 4:**
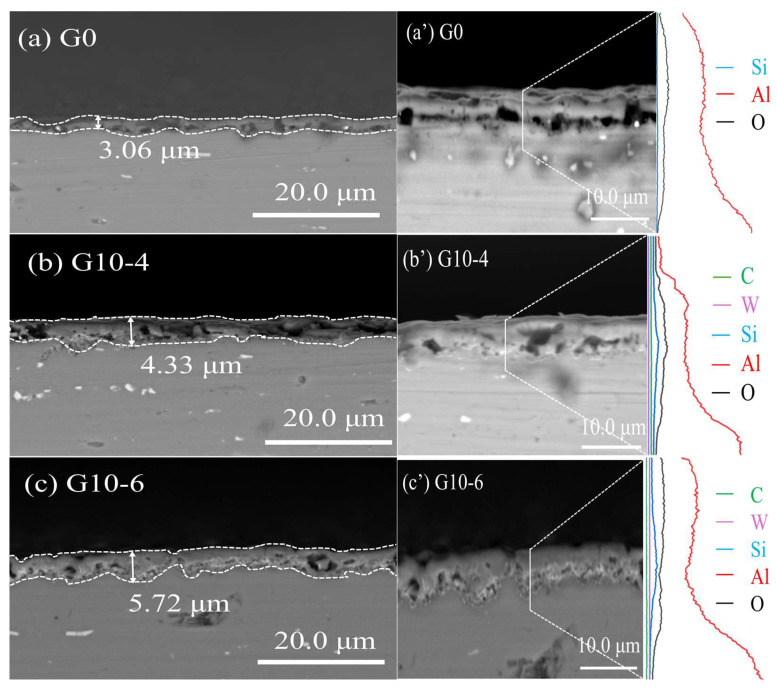

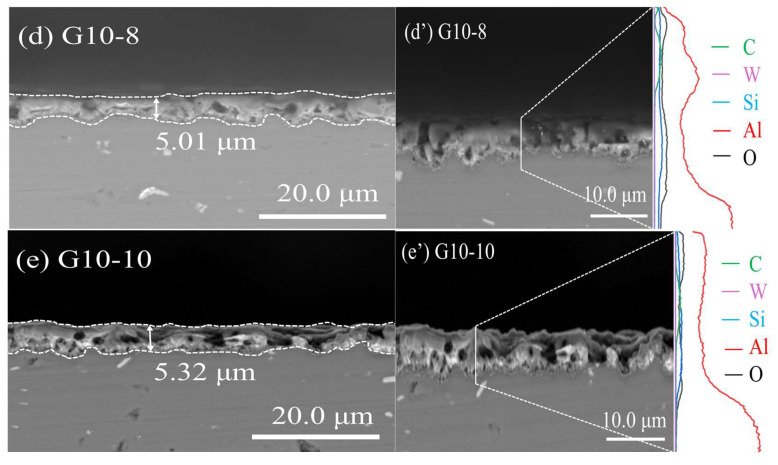
Cross-section morphology and line scanning of MAO coatings with different densities.

Figure 3 shows the cross-sectional morphology and elemental distribution of MAO coatings treated with graphene oxide dispersion and different concentrations of Na_2_WO_4_ solution. From the results of the corresponding EDS spectra, it can be seen that O, Si, and a small amount of C and W elements are enriched in the aluminium-deficient region, indicating that the composite additives of C and W elements penetrate into the defects of the MAO ceramic film. Figure 4 shows the cross-sectional morphology of the micro-arc oxidised coatings with different concentrations of graphene oxide dispersion and Na_2_WO_4_ composite additive. From the figure, it can be seen that both micro-arc oxidation ceramic films have no obvious external loose or internal dense layer. As shown in Figure 4a, the thickness of the blank MAO layer is 3.06 μm. The blank MAO layer contains a large number of micropores and defects, as well as voids between the substrate and the film layer, which are caused by the instantaneous breakdown of the ceramic layer by the micro-arc discharge. Figure 4b shows that, when 10 mL of graphene oxide and 4 g of Na_2_WO_4_ solution are added, the film thickness increases to 4.33 μm and the black voids turn grey. Figure 4c shows that the thickness of the ceramic layer prepared by G10-6 increased by 2.66 μm compared to G0, with a significant increase in the density of the ceramic layer [19]; these increases help to improve the resistance to corrosive ions penetrating into the ceramic structure, leading to a significant increase in corrosive resistance. As shown in Figure 4d,e, excessive amounts of graphene oxide and Na_2_WO_4_ cause a decrease in the internal density of the ceramic layer with the thicknesses of 5.01 μm and 5.32 μm, respectively, and the formation of voids between the substrate and the coating. This phenomenon occurs because the high concentration of Na_2_WO_4_ prevents the carbon in the graphene oxide from adhering effectively to the loose coating. At the same time, the penetration of excess ${WO}_{4}^{2-}$ ions into these pores leads to an increase in the pore size within the dense region, resulting in further loosening of the coatings. Based on the line scans in Figure 4a’–e’, it can be seen that the aluminium (Al) peak presents the maximum intensity, followed by oxygen (O), confirming that Al and O predominantly form this coating material. With the combination of graphene oxide dispersion and Na_2_WO_4_, a carbon-enriched region is formed on the upper surface area; this enrichment is attributed to the rapid solidification of the molten oxide ejected from the discharge channel under the electrolyte-induced “cold quenching” effects, which simultaneously encapsulates the adsorbed graphene on the surfaces of the samples; however, no obvious tungsten (W)-enriched region is observed in the ceramic layer.

### 3.3. Corrosion Resistance of Composite Coatings

**Figure 5:**
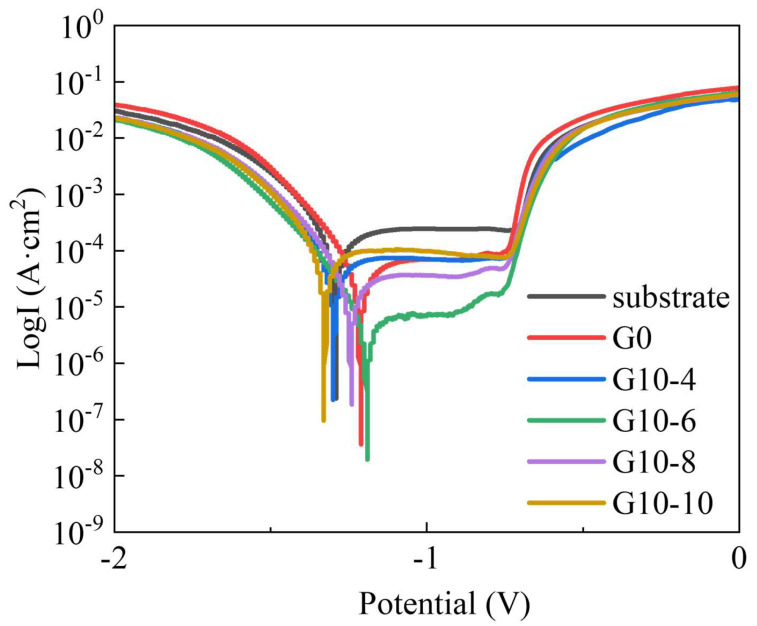
Potential polarisation curves of 6063 aluminium alloy and micro-arc oxidation coatings with different concentrations.

**Figure 6:**
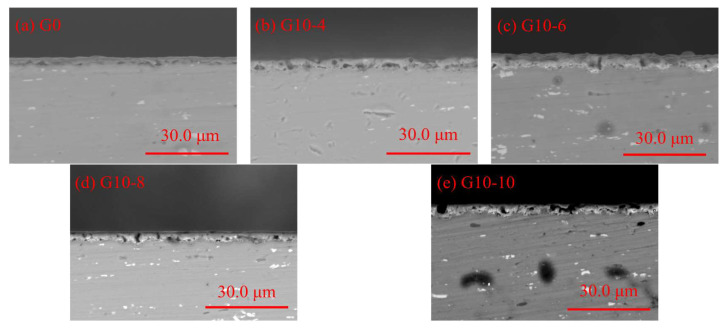
Cross-section morphology after potentiodynamic polarisation.

Figure 5 shows the dynamic potentiodynamic polarisation curves of the MAO coating in 3.5 wt.% solution as a function of concentration. Table 3 summarises the fitting parameters associated with these polarisation curves. Figure 6 shows the cross-sectional morphology according to the dynamic potentiodynamic polarisation curves. As can be seen from the figure, the cross-sectional morphology of the coating did not change much after the dynamic potentiodynamic polarisation curves and no corrosion occurred. As shown in Figure 5 and Table 3, the corrosive current (I_corr_) of the unmodified MAO layer was approximately 2.15 × 10^−5^ A·cm^−2^, and the corrosive potential (E_corr_) was about −1.2137 V. The addition of different concentrations of graphene oxide and Na_2_WO_4_ solutions shifted the self-corrosion potential (E_corr_) of the coating forward, and the self-corrosion current density (I_corr_) was significantly reduced. The G10-6 coating demonstrated the best dynamic potential characteristics, with an I_corr_ value of about 3.01 × 10^−6^ A·cm^−2^, which was one order of magnitude lower than that of the G0 coating, and the E_corr_ shifted to −1.1941 V, which exhibited the lowest corrosion rate (CR), indicating excellent corrosion resistance.

**Figure 7:**
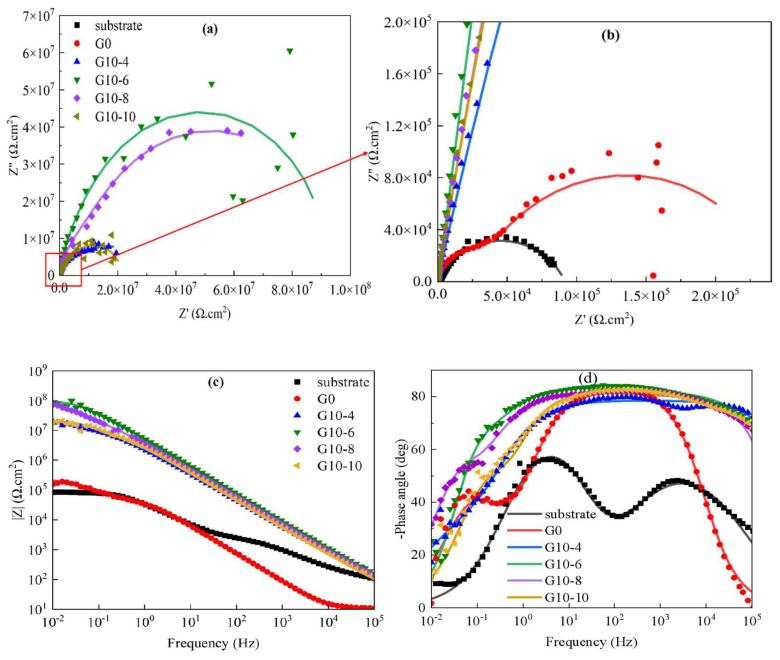
The electrochemical impedance test results of 6063 aluminium alloy and MAO coatings with different concentrations were as follows: (**a**,**b**) Nyquist diagram; (**c**) |Z|-frequency chart; (**d**) phase-angle-frequency diagram.

**Figure 8:**
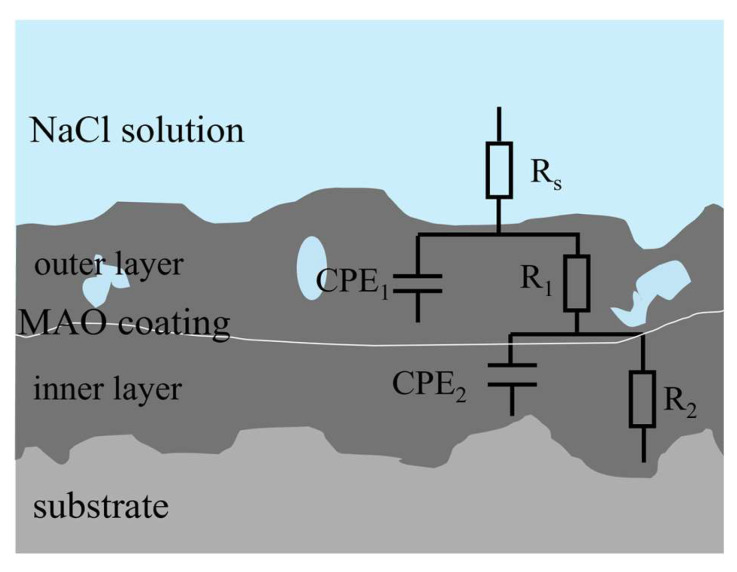
Equivalent circuit diagram.

Figure 7 illustrates the EIS plots for different concentrations of MAO coatings in a 3.5 wt.% sodium chloride solution environment. The corresponding equivalent circuit model diagrams and fitted electrochemical parameters are shown in Figure 8 and Table 4, where R_s_ represents solution resistance, R_1_ denotes the outer layer resistance, R_2_ indicates the inner layer resistance, CPE_1_ represents the external capacitance, and CPE_2_ represents the internal capacitance, respectively. As shown in Figure 6, the capacitive arc diameter of the blank MAO film is the smallest; moreover, the phase angle analysis reveals three different time constants, which reflect not only the resistive properties of the micro-arc oxide film but, also, the interfacial bilayer information between micro-arc oxide and substrate interfaces. The equivalent circuit diagram is shown in Figure 7. In the |Z|-frequency plot, it can be noticed that the low-frequency impedance value |Z| of the MAO coating is at its lowest point, which is only measured at about 8.4 × 10^6^ Ω·cm^2^. In contrast, the G10-6 composite has the largest semicircular arc diameter and a higher impedance film impedance value. In the two observable time constants shown in Figure 6d, the data presented characterise information relating to the composite film layer and the MAO/composite film layer, indicating that corrosion has not yet occurred. The maximum low-frequency impedance modulus was 1.14 × 10^8^ Ω·cm^2^, which is two orders of magnitude higher than that of the blank micro-arc oxide film, and the coatings are more resistant to corrosion. It is consistent with the kinetic potential polarisation curve. The capacitive arc diameters of the G10-8 and G10-10 composite coatings tended to decrease within the range of the Nyquist curves, and their |Z| also decreased to 3.6 × 10^6^ Ω·cm^2^ and 1.7 × 10^7^ Ω·cm^2^, respectively. The Bode plots show the two time constants. The capacitive arc diameter and |Z| were reduced compared to the coating with G10-6 composite solution. The results indicate that the corrosive solutions penetrated into the micro-arc oxide film/substrate interface through cracks and holes in the micro-arc oxide layer in the film/substrate interface, leading to coating degradation.

**Figure 9:**
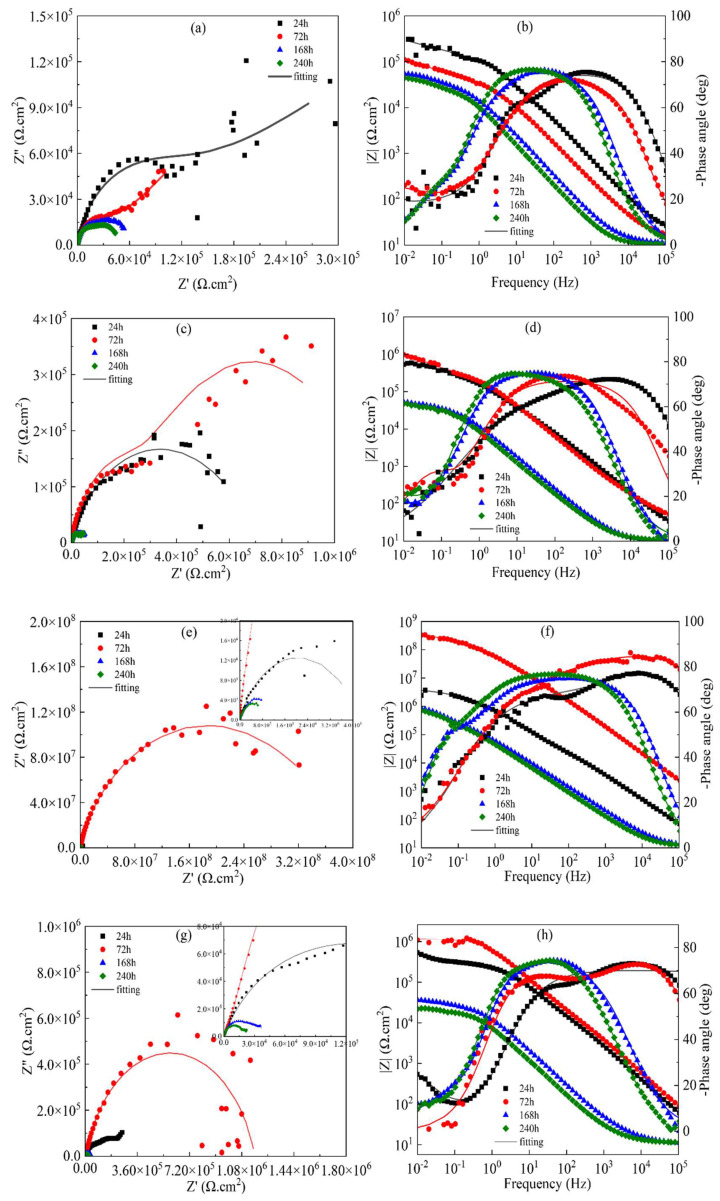

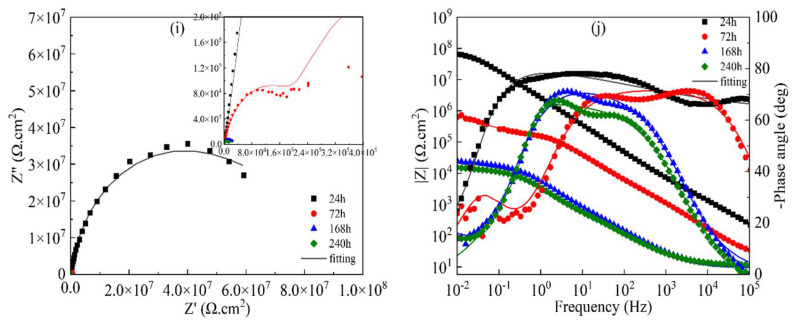
EIS results for MAO coatings immersed in a 3.5 wt.% sodium chloride solution for 24, 72, 168, and 240 h: (**a**,**b**) G0; (**c**,**d**) G10-4; (**e**,**f**) G10-6; (**g**,**h**) G10-8; (**i**,**j**) G10-10.

**Figure 10:**
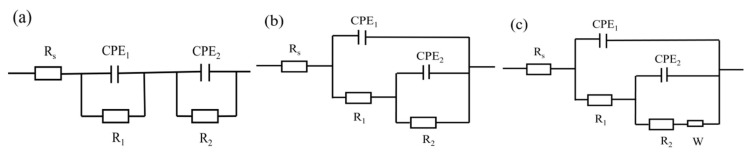
Fitted equivalent circuit diagram: (**a**) G0, (**b**) G10-4, G10-6, (**c**) G10-8, G10-10.

Figure 9a,b show the Nyquist and Bode curves for 24 h, 72 h, 168 h, and 240 h of immersion in 3.5 wt.% NaCl solution. Figure 10a shows the equivalent circuit schematic diagram, where Rs represents the solution impedance, R_1_ represents the micro-arc oxidation film resistance, and R_2_ represents the charge transfer resistance. As shown in Figure 8, the blank-MAO coating has the highest |Z|_0.01Hz_ after immersion in 3.5 wt.% NaCl solution for 24 h. The Bode plots indicate the existence of two time constants characterising the information of the micro-arc oxide film and the bilayer at the oxide/substrate interface, respectively. The results show that the solution enters the film/aluminium substrate interface through cracks and holes in the micro-arc oxide and corrosion starts to occur at the interface. Its corrosion resistance reaches the optimum level. As the immersion time increases, the diameter of the capacitive arc semicircle in the Nyquist diagram increases and the oxide film continues to be damaged. After 240 h of immersion, the |Z|_0.01Hz_ of the coating decreases to approximately 10^4^ Ω·cm^2^, with the worst corrosive resistance.

Figure 9c,d illustrates the Nyquist and Bode plots for 10 mL of G0 and 4 g of Na_2_WO_4_ coating immersed in 3.5 wt.% sodium chloride solution for 24 h, 72 h, 168 h, and 240 h. Figure 10b shows the equivalent circuit simulation diagram, where R_s_ represents the solution impedance, R_1_ represents the micro-arc oxide film resistance, and R_2_ represents the charge transfer resistance. The Bode diagram reveals that the electrochemical response spectrum has two different time constants characterising the information of the micro-arc oxide film and the oxide film/substrate interface bilayer, respectively. This indicates that, during this period, the NaCl solution penetrates between the membrane and the substrate through cracks and holes in the oxide film, leading to corrosion of the membrane. The corrosion resistance of the coating decreases with increasing immersion time, and the |Z|_0.01Hz_ of the coating decreases to 10^4^ Ω·cm^2^ after 240 h of immersion.

The Nyquist plots and Bode plots of 10 mL of graphene oxide and 6 g of Na_2_WO_4_ coatings, which were immersed in 3.5 wt.% sodium chloride solution for 24 h, 72 h, 168 h, and 240 h, are given in Figure 9e,f. The equivalent circuit simulation is given in Figure 10b. As can be seen from the figures, the radius of curvature of the Nyquist plot curve at 72 h is significantly larger than that of the curves at other time periods, and the radius of curvature gradually decreases with the extension of the immersion time. Concurrently, the |Z|-frequency curve in the Bode diagram shows a decreasing trend as a whole; however, |Z|_0.01Hz_ is still as high as 10^6^ Ω·cm^2^. These results show that the micro-arc oxidation coating layer composed of G10-6 has a good corrosion resistance and protective performance.

Figure 9g,j present the Nyquist and Bode plots of G10-8 and G10-10 coatings immersed in 3.5 wt.% sodium chloride solution for 24 h, 72 h, 168 h, and 240 h, respectively. Figure 10c shows a schematic of the equivalent circuit simulation, where R_s_ denotes the solution resistance, R_1_ represents the micro-arc oxide film resistance, R_2_ represents the charge transfer resistance, and W signifies the Warburg diffusion impedance. Coatings G10-8 and G10-10 were immersed in 3.5 wt.% sodium chloride solution for 72 h and 24 h, respectively. It was observed that the low-frequency impedance modulus |Z|_0.01Hz_ reached its maximum value. However, the low-frequency impedance film value |Z|_0.01Hz_ continued to decrease with a further increase in immersion time, indicating gradual degradation of the coating integrity. After 240 h of immersion, both G10-8 and G10-10 coatings showed induced reactance in the low-frequency region; at the same time, the low-frequency impedance film value |Z|_0.01Hz_ decreased to approximately 10^4^ Ω·cm^2^. This indicates that the corrosion of the solution has begun to significantly affect the substrate and, at the same time, significantly reduced the corrosion resistance of the coatings.

**Figure 11:**
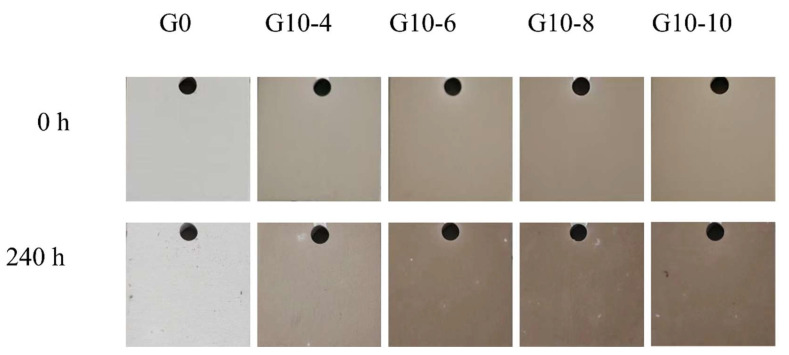
The macroscopic appearance of the MAO coatings immersed in 3.5 wt.% NaCl solution at different concentrations for 240 h.

**Figure 12:**
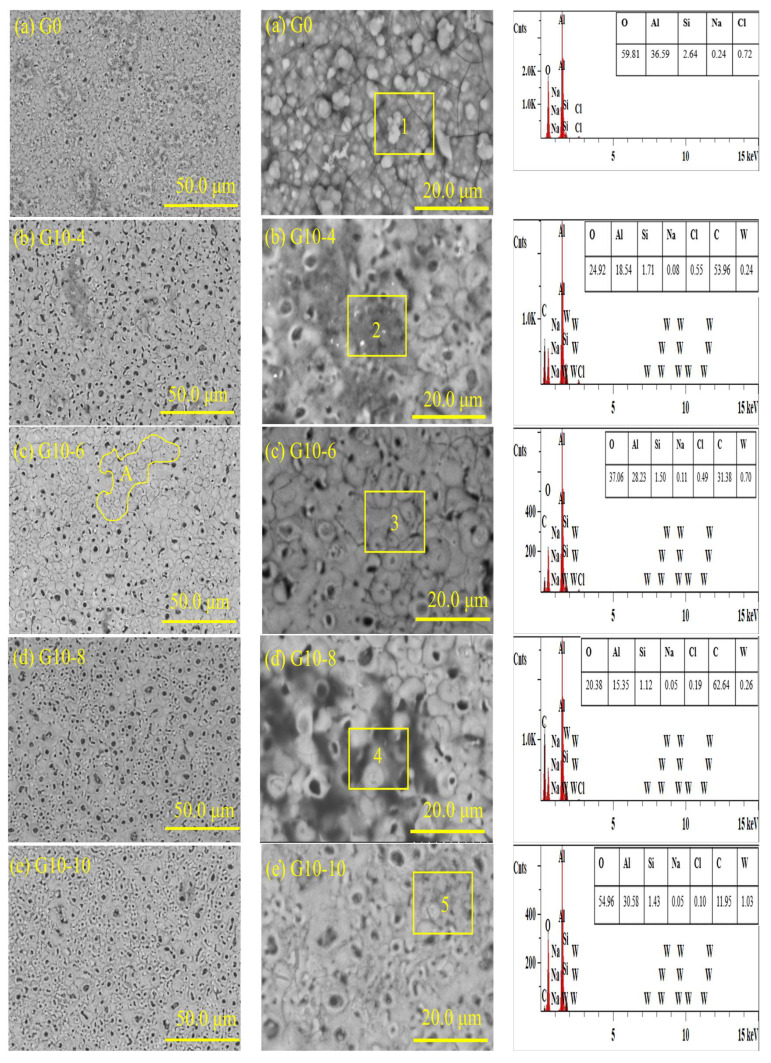
Microstructure of MAO coatings in 3.5 wt.% NaCl solution at different concentrations for 240 h.

Figure 11 shows the macroscopic appearance of MAO coatings immersed in different concentrations of 3.5 wt.% sodium chloride solution for 240 h. As shown in the figure, without immersion, the surface of each specimen was smooth and free of rust spots. Immersed in 3.5 wt.% brine for 240 h, the results showed that a large number of black spots existed on the surfaces of G10-4 and G10-10. Figure 12 shows the high- and low-magnification microscopic morphology and EDS analysis of the thin-film layers with different concentrations. As can be seen from the figure, after 240 h of immersion, corrosion started to appear in regions 1, 2, 4, and 5 of samples G0, G0-4, G10-8, and G10-10 (Figure 12a,b,d,e). This is mainly because the Nacl solution corroded the surface of the coatings, which exposed the C elements attached to the outer layer, and black corrosion areas appeared. From the EDS results, it can be seen that the main components of the coating on the surface of G0 (Figure 12a’) are O and Al, and the main component of the black material on the surface of G10-4 (Figure 12b’) is C, which is due to the corrosion and detachment of the outermost film. The results of G10-8 (Figure 12d’) show that the coating surface has a high content of C of 62.64%, which indicates that corrosion is the most serious. The corrosion on the surface of G10-10 (Figure 12d’) is the most serious. Pitting corrosion occurred on the surface (Figure 12e’). However, on the surface of G10-6 (Figure 12c,c’), no corrosion area was found in area 3. This indicates that, by immersing for 240 h, G10-6 shows better corrosion resistance.

### 3.4. Mechanism Analysis

**Figure 13:**
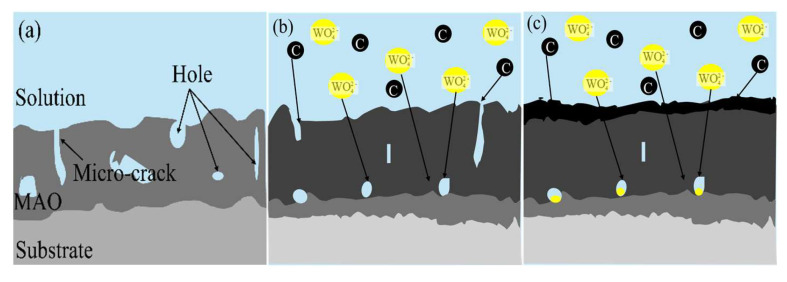
Schematic diagram: (**a**) micro-arc oxidation; (**b**) G0+ Na_2_WO_4_ compound addition; (**c**) appropriate concentration.

Figure 13 shows the mechanism of the composite coating. As shown in Figure 13a, when 6063 aluminium alloy was treated by micro-arc oxidation, the microcracks, holes, and other defects generated during the micro-arc oxidation process were an important pathway for the corrosive medium to diffuse into the film, which reduced the protective effect of the micro-arc oxidised film and led to the corrosion of the substrate. However, when a composite solution of graphene oxide dispersion and Na_2_WO_4_ was added (Figure 13b), element C in graphene oxide and element W in Na_2_WO_4_ entered the cracks and holes of the film under high temperature and pressure. As shown in Figure 13c, the appropriate concentration of the composite additives can effectively fill the microcracks and cavities of the film and improve the densification and corrosion resistance of the film. This is because element C in graphene oxide dispersion and element W in the Na_2_WO_4_ solution can not only be adsorbed on the surface of the coating and substrate as a single element but also exist in the film in the form of mixed adsorption. Notably, graphene enhances the conductivity of the solution [28]. It has been shown [32] that sp^2^ allotropes of carbon materials can produce intrinsic diffusion passivation layers [33,34], and both single and multilayer graphene oxide films coated on metal surfaces can effectively prevent the oxidation of metals. In aqueous solution, the graphene oxide surface contains many anion-containing oxygen-containing functional groups [35], and there is electrostatic repulsion between graphene oxide and anions, which prevents the anions from diffusing to the metal surface for galvanic corrosion [36]. Meanwhile, the presence of ${WO}_{4}^{2-}$ ions in the electrolyte not only participates in the formation of the film but also affects the micro-morphology of the film. An appropriate amount of sodium tungstate also slows down the deposition rate of ${SiO}_{3}^{2-}$ [37]. During the MAO process under a strong electric field, ${WO}_{4}^{2-}$ is easily adsorbed on the surface of the anodic aluminium alloy substrate. This increases the number of charged particles in the plasma electrolytic oxidation reaction with oxygen in the electrolyte solution, which reduces the discharge energy per ion while increasing the discharge channels on the film surface. The subsequent condensation process leads to smaller diameter but more discharge pores, resulting in a smoother, flatter, and denser film. The potential chemistry is shown below [22]:

2WO_4_^2−^ + 4e → 2WO_3_ + O_2_↑ (2)

5WO_4_^2−^ + 2Al → 5WO_3_ + Al_2_O_3_ + O_2_↑ (3)

4Al + 3O_2_ → 2Al_2_O_3_ (4)

Due to the abundance of Al in the discharge channel, the resulting WO_3_ reacts with Al at high temperatures to form monomeric W [22] and releases a large amount of heat. The chemical reactions are as follows [38]:

2WO_3_ + 4Al → 2Al_2_O_3_ + 2W + Q (5)

As the concentration of sodium tungstate increases, the transformation of ${WO}_{4}^{2-}$ into elemental tungsten within the film layer is the main reason for the gradual darkening of the film colour.

## 4. Conclusions

In this study, a micro-arc oxide coating was prepared using graphene oxide and Na_2_WO_4_ as additives to improve the corrosion performance of the micro-arc oxide coating. The conclusions are as follows:

(1) The results of energy spectrum analysis showed that both carbon (C) and tungsten (W) elements in the co-diffusion MAO coating were involved in the reaction of the coating. C is involved in the reaction of the outer layer of the coating and W is also involved in the reaction of the inner layer of the coating, and there is a mutual relationship between W and C.
(2) The corrosion resistance assessment showed that the impedance film value |Z| of the coating with the addition of G10-6 reached an impressive 1.14 × 10^8^ Ω·cm^2^, while the impedance film value of the black MAO coating was only 8.4 × 10^6^ Ω·cm^2^, thus indicating an increase of two orders of magnitude in the value of the impedance film due to the composite addition. After immersion in 3.5 wt.% (mass fraction) brine for 240 h, the corrosive resistance of the G10-6 coating was close to 10^6^ Ω·cm^2^. In addition, no corrosion spots were observed on the G10-6 coating, indicating excellent long-term corrosion resistance.

## Figures and Tables

**Table 1:** Chemical composition (%) of 6063 Al alloy.

Si	Fe	Cu	Mn	Mg	Cr	Zn	Ti	Al
0.39	0.25	0.01	0.02	0.76	0.02	0.001	0.03	98.519

**Table 2:** EDS results from distinct regions.

Point	Al (wt.%)	O (wt.%)	Si (wt.%)	C (wt.%)	W (wt.%)
1	63.60	34.17	2.24	—	—
2	53.74	41.71	3.13	—	3.12
3	36.73	48.01	5.59	9.68	—
4	37.68	57.10	2.62	4.74	—
5	52.56	41.95	3.89	—	4.58
6	44.54	49.94	3.36	—	7.37
7	36.78	57.86	3.44	9.84	—
8	51.05	42.23	4.35		6.30
9	39.24	56.46	3.61	5.47	

**Table 3:** Fitting data of 6063 aluminium alloy and different concentrations of MAO coating PDP.

Samples	b_a_ (mV·dec^−1^)	−b_c_ (mV·dec^−1^)	E_corr_ (V)	I_corr_ (A·cm^−2^)	CR (mm·a^−1^)
substrate	727.33	445.2	−1.2927	8.57 × 10^−5^	1.6814
G0	496.24	515.27	−1.2137	2.15 × 10^−5^	0.42243
G10-4	594.92	498.37	−1.2969	1.77 × 10^−5^	0.34759
G10-6	465.49	560.88	−1.1941	3.01 × 10^−6^	0.059164
G10-8	535.28	519.15	−1.2433	1.10 × 10^−5^	0.21612
G10-10	509.63	683.75	−1.3249	1.17 × 10^−5^	0.23006

**Table 4:** Determination of electrochemical parameters of micro-arc oxidation coatings with different composite additives by EIS method.

Samples	R_s_	R_1_	CPE_1_		R_2_	CPE_2_		R_t_	Chi-Squared
	Ω·cm^2^	Ω·cm^2^	Y_1_ Ω^−1^∙s^n^∙cm^−2^	n_1_	Ω·cm^2^	Y_2_ Ω^−1^∙s^n^∙cm^−2^	n_2_	Ω·cm^2^	
substrate	10.79	49,970	3.25 × 10^−6^	0.93	1.89 × 10^5^	2.02 × 10^−5^	0.84	2.4 × 10^5^	7 × 10^−3^
G0	12.78	25,207	1.46 × 10^−7^	0.86	8.4 × 10^6^	2.43 × 10^−7^	0.48	8.4 × 10^6^	9 × 10^−3^
G10-4	13.32	9 × 10^5^	7.94 × 10^−8^	0.87	2.27 × 10^7^	2.20 × 10^−7^	0.60	2.37 × 10^7^	3 × 10^−3^
G10-6	40.31	1 × 10^6^	1.88 × 10^−7^	1.12	1.13 × 10^8^	3.99 × 10^−8^	0.90	1.14 × 10^8^	9 × 10^−3^
G10-8	10.2	2027	0.39 × 10^−7^	0.86	3.57 × 10^6^	1.91 × 10^−6^	0.93	3.6 × 10^6^	4 × 10^−3^
G10-10	9.8	2 × 10^6^	6.61 × 10^−8^	0.91	1.40 × 10^7^	1.97 × 10^−7^	0.67	1.7 × 10^7^	3 × 10^−3^

## Data Availability

The original contributions presented in this study are included in the article. Further inquiries can be directed to the corresponding author.
